# Mood Sensitive Stocks and Sustainable Cross-Sectional Returns During the COVID-19 Pandemic: An Analysis of Day of the Week Effect in the Chinese A-Share Market

**DOI:** 10.3389/fpsyg.2021.630941

**Published:** 2021-02-26

**Authors:** Qurat ul Ain, Tamoor Azam, Tahir Yousaf, Muhammad Zeeshan Zafar, Yasmeen Akhtar

**Affiliations:** ^1^School of Public Finance and Taxation, Southwestern University of Finance and Taxation, Chengdu, China; ^2^School of Management and Economics, Kunming University of Science and Technology, Kunming, China; ^3^Department of Business Studies, Business School of Bahria University, Karachi, Pakistan; ^4^Department of Management Sciences, University of Central Punjab, Lahore, Pakistan; ^5^Noon Business School, University of Sargodha, Sargodha, Pakistan

**Keywords:** Quality Minus Junk strategy, COVID-19, speculative stocks, mood, sustainable cross-sectional Returns

## Abstract

This study examines two stock market anomalies and provides strong evidence of the day-of-the-week effect in the Chinese A-share market during the COVID-19 pandemic. Specifically, we examined the Quality minus Junk (QMJ) strategy return on Monday and FridayQuality stocks mean portfolio deciles that earn higher excess returns. As historical evidences suggest that less distressed/safe stocks earn higher excess returns (Dichev, 1998).. The QMJ factor is similar to the division of speculative and non-speculative stocks described by Birru (2018). Our findings provide evidence that the QMJ strategy gains negative returns on Fridays for both anomalies because the junk side is sensitive to an elevated mood and, thus, performs better than the quality side of portfolios on Friday. Our findings are also consistent with the theory of investor sentiment which asserts that investors are more optimistic when their mood is elevated, and generally individual mood is better on Friday than on other days of the week. Therefore, the speculative stocks earned higher sustainable stock returns during higher volatility in Chinese market due to COVID-19. Intrinsically, new evidence emerges on an inclined strategy to invest in speculative stocks on Fridays during the COVID-19 pandemic to gain sustainable excess returns in the Chinese A-share market.

## Introduction

A significant boost in economic uncertainty was observed after the outbreak of Corona virus (COVID-19) and the consequences of the virus led the world toward a turbulent economy (Baker et al., 2020). To control the spread of the pandemic, the Chinese government has enacted strict measures, such as lockdown, that may have had some negative impact on the economy (Fareed et al., 2020; Shehzad et al., 2020). A substantial impact was observed on the economic activities of the country and economic slowdown was expected. Capital markets are an important part of economic development and, therefore, the Chinese stock markets were also badly influenced by this pandemic. US stock markets have also observed their highest ever levels of volatility (Baker et al., 2020). Though many studies have observed the economic policies (Huang et al., 2020) and economic consequences (Chen et al., 2020) of the pandemic in China, observing the impact of COVID-19 on speculative stocks and its effect on particular days of the week is still an unexplored area of research. Therefore, this study presents an examination of the effect of specific days of the week on young and distress anomalies during the time of Covid-19 in light of the sentiment hypothesis.

Birru (2018) provided a striking pattern of anomaly returns in the U.S. stock market. Specifically, Birru’s (2018) findings explained that the speculative leg of portfolios gains the highest (lowest) returns on Mondays when they fall on the long (short) leg of the anomalies^2^. Speculative stocks are young or distressed stocks or those that are not easy to value, which are suited for speculation and highly affected by investor sentiment. Psychology research predicts that individuals’ moods comparatively improve on Fridays and worsen on Mondays. Hence, the speculative side of stocks tends to outperform (underperform) due to increases (decreases) in moods on Fridays (Mondays). This scenario leads to the day-of-the-week effect in cross-sectional stock returns.

It is difficult for academicians and practitioners to understand all factors that should be considered in relation to the theory of asset pricing (Elton et al., 1998). This phenomenon has been addressed by considering individual and market rationality in the Efficient Market Hypothesis and Capital Assets Pricing Model existing finance theories (Rasheed et al., 2016). An ambiguous, uncertain, volatile, and complex investment environment triggers investors to speculate outcomes and challenge the existing market efficiency assumptions of rational and well-informed investors. In such situations, investors are constrained from cognitive resources and timing effects. The limited capacity to process information results in poor judgment and decision-making. This situation is addressed in behavioral finance research under the three distinct pillars of sentiment, biases, and heuristics (Hirshleifer, 2001). Behavioral finance researchers critique traditional finance theories and argue in favor of the psychological aspect of investors as a core determinant of asset pricing. Sentiments, which are part of human psychology, affect investors’ decision-making both individually and collectively (Peterson, 2016). Therefore, researchers have explored the role of individual and market sentiments in the mispricing of stocks. Research on investor sentiment and its role in explaining cross-sectional variations led researchers to develop divergent viewpoints (Bormann, 2013).

Various scholarly research outcomes discuss the empirical explanations of asset pricing bubbles as external factors of capital markets, like macroeconomic factors (Ying et al., 2019, 2020). These mainly originate from regulatory reforms made to correct the flaws in investment regulation norms. However, factors affecting the asset pricing bubbles cannot solely be observed through the endogenous factors of investor behaviors that lead to excitement or losing confidence over financial markets (Öztürk et al., 2020). This study contributes to the literature: it presents a specific explanation of stocks as being sensitive to sentiment, and it provides evidence of cross-sectional variations of returns on particular days by linking a speculative leg of anomalies with mood theory from psychology literature. This study also provides different investment strategies to earn excess returns across the days of the week by investing in speculative stocks.

Economists have always argued that the decision-making process cannot be properly analyzed in the absence of knowledge about the psychological aspect of an individual’s thought process, as an individual’s thinking is shaped by the co-existence of both internal and external sentimental factors (Hume and Hendel, 1955). Psychology research goes one step further than statistics and traditional economics in its treatment of the process of decision-making under conditions of uncertainty by centering on the nature of the stimulus rather than simply focusing on the outcomes resulting from that stimulus. In order to objectively examine a stimulus, every event that has happened should be considered equally likely to occur, and extreme variations in statistics should be considered outliers to the stimulus. However, this assumption puts finance scholars on the blind path of behavioral responses under varied stimuli by avoiding the psychological facets of information processing and learning (Estes and Burke, 1953). Sentiments facilitate the process of decision-making by influencing information processing and the learning phase (Davidson et al., 2000). The process of learning can be a gradual phase or sometimes can be a sudden occurrence that is dependent upon information processing from a variety of perspectives (Kahneman, 2011).

Therefore, using the QMJ factor developed by Asness et al. (2019), this study explored the cross-sectional variations in speculative anomalies on different days of the week in the Chinese A-share market during the COVID-19 pandemic. The following are our motivations for conducting this study. First, the speculative characteristics of stocks exist at the same time as the peculiarity between junk and quality stocks, according to Asness et al. (2019). The findings of Asness et al. (2019) revealed that data from multiple countries indicated that quality stocks outperformed junk stocks. Quality stocks are those that are easy to value and safe to invest in, whereas junk stocks are those that are not easy to value, are accompanied by investment risks, and are suitable for speculation or have speculative characteristics. Meanwhile, the process of identifying the day-of-the-week effect in QMJ stocks resembles the analysis conducted by Birru (2018). Second, Birru’s (2018) analysis is appropriate for the U.S. market but is an unresolved question for other markets, like China’s, that have unique structures. With the economic development of China, rapid growth was observed in the Chinese stock market, raising it to the distinction of being the second largest stock market in the world. However, regardless of its size, trading patterns in the Chinese stock market are most chaotic amongst emerging markets, with higher volatility and highest and lowest cycles determined by individual investors and huge interference from the government. Chinese firms hold maximum individual shareholding patterns and less institutional shareholding; therefore, it is important to verify this relationship in the Chinese market because individuals are more sensitive to investor sentiment than institutions are. Our third motivation for conducting this investigation is that our findings will contribute an additional explanation for cross-sectional variations to the literature. Asness et al. (2019) provided the explanation that cross-sectional variations occur due to mispricing; our results elaborate on this explanation by connecting the QMJ factor with investor sentiment and making this explanation more specific than those elicited through prior research. Lastly, it is very important to determine that how speculative stocks behave during the pandemic and post-pandemic time period because speculative stocks are prone to sentiment. Therefore, it is expected that speculative stocks should perform better on Fridays due to higher mood and investor sentiment should also be high because stock markets contain higher volatility due to the effect of the pandemic.

## Materials and Methods

In this section, we discuss the data sources and methodology used to calculate portfolios based on anomalies for which the speculative leg exists in the junk factor. Data were obtained from the Wind Database, which is the largest financial database for Chinese data. The time period covered in our analysis is from February 2020 to September 2020, and we targeted the A-share market of China and collected data from both the Shanghai and Shenzhen stock exchanges. We chose the post-January timeframe for data collection for the following reasons. First, we needed to consider the pandemic time period. On January 23, just a day before the Chinese New Year, Wuhan was sealed and the government suspended all Chinese New Year festival activities. Additionally, the government suspended all public gatherings and all schools were closed.

The pandemic also affected economic activities and an economic slowdown was observed in the Chinese economy. On February 3rd, the Chinese stock markets reopened and faced a decline in the index on the first day. Therefore, the pandemic lockdown due to COVID-19 is the main event window of the stock market and it is important for researchers to test the impact of COVID-19 on the speculative stocks on specific days of the week. Although different trading principles were introduced years ago, most companies did not know how to implement them, which created several discrepancies at the time. Another reason we selected the data during COVID-19 is that we imposed the condition to take a minimum number of observations for each portfolio cut point. The analysis of the QMJ effect on different days of the week is similar to the analysis of the anomalies that are sensitive to investor sentiment referred to in Birru’s (2018) work.

In this paper, the QMJ factor is used to analyze the distress anomaly and stocks that are young. The distress anomaly was measured following Ohlson’s (1980) O-Score method, and the age anomaly was measured using the method from Baker and Wurgler, 2006) [Sections “Anomaly 1: Distress (O-Score)” and “Anomaly 2: Age” elaborates on both anomalies]. By following these methods for each anomaly, 10 equal deciles were generated for the purpose of analyzing portfolios. Further, we considered only the first and tenth deciles of each anomaly because quality stocks are those that fall in the tenth decile and junk stocks fall in the first decile of each anomaly. Portfolios based on both anomalies are rebalanced annually. Therefore, the first decile of all anomalies was predicted to perform better, as the speculative leg exists in the junk side of the anomaly. Moreover, a new strategy emerged with the prediction of higher QMJ returns on Mondays compared to Fridays because of the existence of the speculative leg in the junk side of the anomalies. Details of the anomalies and predicted returns are provided in Table 1.

**TABLE 1:** Description of anomalies and speculative investment strategies.

	**Age anomaly**	**O-score anomaly**
Quality stocks	Decile 10	Decile 1
Junk stocks	Decile 1	Decile 10
Speculative leg	Decile 1	Decile 10
Predicted quality minus junk strategy returns on Friday	Lower	Lower
Predicted quality minus junk strategy returns on Monday	Higher	Higher
Predicted speculative leg return on Friday	Higher	Higher
Predicted speculative leg return on Monday	Lower	Lower
Explanation (why speculative)	Young stocks	Close to distress

*The table describes the division of samples for anomalies and speculative strategies. It indicates the division of anomalies into Quality and Junk stocks, and also indicates the expected speculative leg for each anomaly and offers a brief explanation for speculative reasons. Table 1 also reports the expected returns for speculative leg on a particular day.*

### Anomaly 1: Distress (O-Score)

Financially distressed stocks are sensitive to sentiment and highly affected by sentiment because higher distressed stocks are more risky and riskier stocks are prone to sentiment. The variation in sentiment will have a contemporary effect on returns and highly affect the prices of stocks that are not easy to value or very subjective to value or are difficult to arbitrage (Baker and Wurgler, 2006).

According to Dichev (1998), more highly distressed firms outperform stocks that are not distressed. Therefore, speculative characteristics fall in the junk side of the anomaly, so the predicted QMJ returns should be greater on Mondays than on Fridays. The speculative leg should perform better on Fridays and have a reversal effect on the QMJ factor.

Ohlson’s (1980) O-Score model is measured as

(1) -1.32-0.407⁢log⁡(T⁢A)+6.03⁢T⁢L⁢T⁢A-1.43⁢W⁢C⁢T⁢A+0.076⁢C⁢L⁢C⁢A-1.720⁢E⁢N⁢E⁢G-2.37⁢N⁢I⁢T⁢A-1.83⁢F⁢U⁢T⁢L+0.285⁢I⁢N⁢T⁢W⁢O-0.521⁢C⁢H⁢I⁢N

Here in Eq. 1, TA denotes Total Assets, while TLTA represents the leverage ratio and comprises the book value of Total Debt to Total Assets. WCTA is the ratio of Total Working Capital divided by Total Assets. CLCA refers to the inverse of the liquidity ratio and is measured by Current Liabilities over Current Assets. If the value of Total Debt is greater than the value of Total Assets, then the ENEG will be 1, and if it is less than the Total Assets, then it will be 0. NITA is the ratio of Net Income to Total Assets and is measured as Net Income divided by Total Assets. FUTL is the ratio of funds received through operations divided by Total Liabilities. INTWO will be 0 if the Net Income is positive in either of the 2 previous years, and it will be 1 if the Net Income is negative for the last 2 consecutive years. CHIN is estimated by (*NI*_*t*_−*NI*_*t*−1_)/(|*NI*_*t*_| + |*NI*_*t*−1_|), and here NI denotes Net Income.

### Anomaly 2: Age

Stocks that are comparatively young are sensitive to sentiment. Moreover, young stocks will be most affected by sentiment.

Historic evidence suggests that old stocks earn higher returns than young stocks. For example, in the long run, Initial Public Offerings tend to underperform (Ritter, 1991). Therefore, older stocks are classified as quality stocks and young stocks are classified as junk stocks, according to the QMJ strategy. For the age anomaly, the speculative leg is in the junk side of the strategy and predicts that QMJ strategy returns will be higher for Mondays than for Fridays.

Age is calculated using the Baker and Wurgler (2006) method, where age is the number of months since the firm appeared in the Chinese stock market. For the age anomaly, portfolios are rebalanced annually at the end of December.

## Results and Discussion

### QMJ Strategy Returns

Table 2 (Panels A and B) presents weekly alpha values for QMJ strategy returns in the presence of different asset pricing models for Fridays and Mondays during the COVID-19 period. The findings are consistent for both anomalies on Fridays because Fridays earn negative QMJ strategy returns, as the speculative leg falls in the junk side of the anomaly. Fridays alone account for 109 basis points in excess of returns for young stocks than for older stocks, and distress stocks also earn 66 basis points in excess of returns for distressed stocks than for non-distressed stocks on Fridays. Therefore, findings are consistent for both anomalies based on the theory that Fridays should see higher returns for the speculative leg of the anomaly than for the non-speculative leg. Our results are also consistent when we compare return patterns across days. Fridays earn a higher magnitude return for both anomalies than Mondays. Furthermore, our findings are consistent with the theory of investor sentiment and the psychology literature that finds that junk stocks perform better on Fridays because of happier moods and worsen on Mondays due to lower moods. Additionally, the findings are also consistent with the explanation that speculative stocks perform better on Fridays during the risky time period and Chinese stock markets faced higher risk during the COVID-19 time period.

**TABLE 2:** Monday and Friday quality minus Junk strategy returns.

**Panel A: Friday quality—junk**				
	**CAPM**	**FF3**	**Carhart4**	**FF5**
Age anomaly	−0.0109195	−0.0105443	−0.0098037	−0.0096892
(t statistics)	(−3.71)	(−3.52)	(−3.22)	(−3.14)
O-score anomaly	−0.0066307	−0.0071671	−0.0063236	−0.0047223
(t statistics)	(−3.23)	(−3.44)	(−3.02)	(−2.35)

**Panel B: Monday quality—junk**				
	**CAPM**	**FF3**	**Carhart4**	**FF5**
Age anomaly	−0.0052552	−0.005139	−0.0047982	−0.0038774
(t statistics)	(−5.22)	(−5.02)	(−4.64)	(−3.89)
O-score anomaly	−0.0025017	−0.002875	−0.0025607	−0.0023636
(t statistics)	(−5.25)	(−6.14)	(−5.54)	(−5.21)

*Table 2 examines Quality Minus Junk strategy returns of the portfolios prepared on the basis of Age anomaly and O score anomaly to invest on Friday and Monday. Panel A represents QMJ weekly returns on Friday and panel B represents QMJ weekly returns on Monday. Both panels consist of the alpha values based on the Fama and French 5 factor model, Carhart 4 factor model, Fama and French 3 factor Model, and the Capital Assets Pricing Model. Portfolios are generated on the basis of Value weighted technique and alpha values are also adjusted for heteroskedasticity and autocorrelation.*

### Friday Minus Monday

Table 3 provides a direct analysis of Fridays and Mondays, indicating that investors earn higher returns on Mondays when Monday’s QMJ strategy returns are adjusted/deducted from Friday’s QMJ strategy returns. The negative alpha values for age and O-Score anomalies from Panel C of Table 3 again verified our results that Mondays see higher QMJ returns than Fridays because speculation characteristics fall in the junk side of the anomalies. Therefore, the Friday minus Monday strategy returns contain negative alpha values in order to provide a robust explanation for investor sentiment on a particular day. The results for age and O-Score anomalies are consistent with the mood theory that Mondays gain higher QMJ strategy returns in comparison to Fridays because moods are higher on Fridays than on Mondays and speculative characteristics fall in the junk side of the anomalies.

**TABLE 3:** Friday minus Monday strategy returns.

**Panel A: Friday (quality—junk)**				
	**CAPM**	**FF3**	**Carhart4**	**FF5**
Age	−0.0109195	−0.0105443	−0.0098037	−0.0096892
(t statistics)	(−3.71)	(−3.52)	(−3.22)	(−3.14)
O score	−0.0066307	−0.0071671	−0.0063236	−0.0047223
(t statistics)	(−3.23)	(−3.44)	(−3.02)	(−2.35)

**Panel B: Monday (quality—junk)**				
	**CAPM**	**FF3**	**Carhart4**	**FF5**
Age	−0.0052552	−0.005139	−0.0047982	−0.0038774
(t statistics)	(−5.22)	(−5.02)	(−4.64)	(−3.89)
O score	−0.0025017	−0.002875	−0.0025607	−0.0023636
(t statistics)	(−5.25)	(−6.14)	(−5.54)	(−5.21)

**Panel C: Friday—Monday**				
	**CAPM**	**FF3**	**Carhart4**	**FF5**
Age	−0.0056642	−0.0054053	−0.0051378	−0.0051378
(t statistics)	(−1.98)	(−1.85)	(−1.73)	(−1.73)
O score	−0.004129	−0.0042921	−0.0037629	−0.0023588
(t statistics)	(−2.02)	(−2.07)	(−1.79)	(−1.14)

*Table 3 examines Quality Minus Junk strategy returns of the portfolios prepared on the basis of Age anomaly and O score anomaly to invest on Friday and Monday. Panel A represents QMJ weekly returns on Friday, panel B represents QMJ weekly returns on Monday, and panel C represents the QMJ strategy returns for Friday minus Monday. All panels consist of the alpha values based on the Fama and French 5 factor model, Carhart 4 factor model, Fama and French 3 factor Model, and the Capital Assets Pricing Model. Portfolios are generated on the basis of Value weighted technique and alpha values are also adjusted for heteroskedasticity and autocorrelation.*

Here, the results also confirm the explanation of the day-of-the-week effect for young and distress anomalies that asserts that junk stocks earn higher returns on Fridays due to the existence of the speculative leg in the junk side of the anomaly, and the speculative leg earns higher returns with higher moods during the COVID-19 period.

### Asymmetric Returns of Junk Side

Panels A, B, and C of Table 4 present the results of the difference in returns in the junk side of Fridays and Mondays. The theory of mispricing based on investor sentiment must provide asymmetric results when returns of the speculative leg are compared for both days. The sentiment-based explanation must be endorsable with the returns trend of the speculative leg. Hence, Panels A, B, and C display only the junk side of the anomalies based on young stocks and distress stocks. The strategy returns of the junk side portfolios for both days are separately given in Panels A and B, while Friday junk minus Monday junk weekly portfolio excess returns are presented in Panel C of Table 4. The results are robust with the explanation that returns should be asymmetric when the speculative leg on Fridays is tested against the speculative leg on Mondays, and our results reconfirm that young and distress stocks are sensitive to investor sentiment. Focusing on the alpha values of both anomalies, the results suggest that the explanation for the difference in returns in the junk side for young stocks and distress stocks (Panel C) is consistent with the sentiment hypothesis, based on investor mood that the day-of-the-week effect prevails in cross-sectional returns for the speculative leg. The literature predicts that the junk side of the anomalies contain the speculative leg; therefore, we only focus on it to verify that returns are asymmetric within the speculative leg across days.

**TABLE 4:** Asymmetric in the speculative leg.

**Panel A: Friday junk**				
	**CAPM**	**FF3**	**Carhart4**	**FF5**
Age	0.0094369	0.0090749	0.0084361	0.0081909
(t statistics)	(3.15)	(2.98)	(2.73)	(2.62)
O score	0.003523	0.003808	0.0030405	0.0016345
(t statistics)	(1.72)	(1.83)	(1.45)	(0.80)

**Panel B: Monday junk**				
	**CAPM**	**FF3**	**Carhart4**	**FF5**
Age	0.0028003	0.0027147	0.0023018	0.0014588
(t statistics)	(2.44)	(2.33)	(1.96)	(1.25)
O score	−0.0000801	0.0001467	−0.0000828	−0.0002737
(t statistics)	(−0.13)	(0.23)	(−0.13)	(−0.42)

**Panel C: Friday junk—Monday junk**				
	**CAPM**	**FF3**	**Carhart4**	**FF5**
Age	0.0066365	0.0063602	0.0059934	0.0067321
(t statistics)	(2.22)	(2.09)	(1.95)	(2.14)
O score	0.0036031	0.0036613	0.0031233	0.0021642
(t statistics)	(1.74)	(1.73)	(1.46)	(1.05)

*Table 4 examines Quality Minus Junk strategy returns of the portfolios prepared on the basis of Age anomaly and O score anomaly to invest on Friday and Monday. Panel A represents Junk portfolio weekly returns on Friday, panel B represents Junk portfolio weekly returns on Monday, and panel C represents the Junk portfolio returns on Friday minus junk portfolio returns on Monday. All panels consist of the alpha values based on the Fama and French 5 factor model, Carhart 4 factor model, Fama and French 3 factor, and the Model and Capital Assets Pricing Model. Portfolios are generated on the basis of Value weighted technique and alpha values are also adjusted for heteroskedasticity and autocorrelation.*

### Robustness Test

#### Macroeconomic News Impact

It is rarely possible for a systematic pattern to be found in the announcement of better or worse news on a specific day of the week, but it is likely that, due to these announcements, a systematic pattern of cross-section returns will be generated against these macro announcements. It is also possible that some anomalies are more sensitive to these announcements than others. Hence, we collected the data of the Producer Price Index and Consumer Price Index on a weekly basis following the methodology of Savor and Wilson (2013). We focused on the dates when these announcements were publicly declared. Panels A, B, and C of Supplementary Table S1 provide results of the strategy returns for all anomalies. The specific return dates of these announcements are omitted from the portfolios. Our results are again robust with the existing explanation for both days that the prevailing variations are due to mood fluctuations, and macroeconomic news does not significantly impact the anomaly returns.

#### Firm Specific News Impact

There is a possibility that existing cross-sectional variations in the anomalies are due to non-random announcements of organization-specific news. Hence, it is necessary for the validation of this argument that non-speculative and speculative stocks are clearly differentiated with regard to good news and bad news. To verify this explanation, we collected the firm-specific data of dividend announcements and earnings announcements and followed the methodology of Dellavigna and Pollet (2009), which recommends eliminating the earning announcement dates. We used a conservative approach for this analysis and omitted 2 days before and 2 days after the declaration. This approach is useful because ignoring these 5 days will not affect any day of the week because the elimination of 5 days will stand the balance of the week. Panels A, B, and C of Supplementary Table S2 present the results of the anomaly returns and exclude the data from the announcement dates. Our results provide a robust explanation for existing variations that organization-specific news did not significantly change the magnitude or nature of existing relationship. A more direct explanation from our results is that cross-sectional variations were not observed as an impact of organization-specific news.

## Discussion

The psychological explanation of greater mood elevation on Fridays than on other days of the week predicts that the portfolios’ returns should be higher on Fridays than on Mondays. The sentiment hypothesis explains that investor sentiment is higher during times of elevated moods for the speculative leg of the anomalies, and psychological research provides several significant findings that indicate higher moods are experienced on Fridays, while lower moods are experienced on Mondays. Therefore, portfolio returns based on both anomalies for the speculative leg should be greater for Fridays than for Mondays. The speculative leg of the anomalies exists in the junk side of both anomalies. The findings are robust for all the asset pricing models, and other models also provide a striking magnitude of excess in returns like those realized for the Capital Assets Pricing Model. Moreover, compared to the distress anomaly, young stocks earn a greater magnitude of returns for weekly portfolios.

The outcomes of direct analysis of Fridays and Mondays indicates that investors earn higher returns on Mondays when Monday’s QMJ strategy returns are adjusted/deducted from Friday’s QMJ strategy returns. The Friday minus Monday strategy returns contain negative alpha values in order to provide a robust explanation for investor sentiment on a particular day. The results are also robust with the explanation that returns should be asymmetric when the speculative leg on Fridays is tested against the speculative leg on Mondays. Moreover, the same findings were attained for both days that the prevailing variations are due to mood fluctuations, and macroeconomic news and Firm Specific news do not significantly impact the anomaly returns.

## Conclusion

This study revealed a substantial anticipated relationship between anomaly returns and different days of the week. Chinese A-share market data from both the Shanghai and Shenzhen stock exchanges were used to evaluate the QMJ factor strategy that resembles the mutual measure of quality (non-speculative/long leg) stocks and junk (speculative/short leg) stocks. The QMJ strategy provided negative anomaly returns on a Friday, which confirmed our findings that junk stocks that also contain the speculative leg perform better than quality stocks due to elevated moods during the COVID-19 period. Our findings are consistent with the work of Birru (2018), who found that short leg/speculative leg stocks are sensitive to investor sentiment, and we have contributed to his findings in a way by testing a new investment strategy for quality and junk stocks in place of the long minus short strategy. The findings are also consistent with a new explanation that a higher risk prevails in Chinese stock markets due to the COVID-19 pandemic and speculative leg of the anomalies perform better on Friday than Monday as literature suggested that speculative stocks perform better on Friday during a higher risk time period. Therefore, we also observed that the QMJ strategy performed in the same way as Birru’s (2018) long minus short strategy. The findings also reveal that the QMJ strategy premium is probably an indicator of behavioral mispricing.

### Limitations and Future Directions

There are several limitations to this study. Firstly, a limited time period has been used for the current study because the post-pandemic time period is short. Therefore, this type of study would have more generalized findings with a larger time span. Secondly, this study has taken data from China, but the pandemic has affected almost the entire world so further studies can take data from several countries on a larger scale. Further, the future studies can test several anomalies to verify the effect of the pandemic on a larger scale.

## Data Availability Statement

The raw data supporting the conclusions of this article will be made available by the authors, without undue reservation.

## Author Contributions

TY performed the formal analysis, the methodology of the manuscript, and technique applications through software. TA analyzed the data. QA conceptualized the idea and reviewed the manuscript. MZ reviewed and edited the draft and also helped in the data collection. YA reviewed the write-up of the manuscript. All authors contributed to the article and approved the submitted version.

## Conflict of Interest

The authors declare that the research was conducted in the absence of any commercial or financial relationships that could be construed as a potential conflict of interest.

## Footnotes

2 Long leg means portfolio deciles that earn higher excess returns, as historical evidence suggests that old age stocks earn higher excess returns (Birru, 2018).
