# Supplementary material for: Mood Sensitive Stocks and Sustainable Cross-Sectional Returns During the COVID-19 Pandemic: An Analysis of Day of the Week Effect in the Chinese A-Share Market
Source: Front Psychol. 2021 Feb 26;12:630941. doi: 10.3389/fpsyg.2021.630941 (PMC7952750; doi:10.3389/fpsyg.2021.630941)
Supplement: Supplementary file 1 [file Table_1.DOCX]

Supplementary Material

## Supplementary Tables

**Table S1: Exclusion of Macroeconomic news announcement dates**

**Panel A: Friday Quality-Junk**

|  | **CAPM** | **FF3** | **Carhart4** | **FF5** |
| --- | --- | --- | --- | --- |
| Age | -.0117364 | -.011549 | -.010823 | -.010958 |
| (t Statistics) | (-3.11) | (-3..02) | (-3.39) | (-3.28) |
| O Score | -.009483 | -.008973 | -.008692 | -.009284 |
| (t statistics) | (-3.12) | (-3.28) | (-2.93 ) | (-2.61) |

**Panel B: Monday Quality-Junk**

|  | **CAPM** | **FF3** | **Carhart4** | **FF5** |
| --- | --- | --- | --- | --- |
| Age | .0019262 | .0011741 | .0009257 | .0007627 |
| (t Statistics) | (3.91) | (4.29) | (4.12) | (3.82) |
| O Score | .0007597 | .0008464 | .0007955 | .0006264 |
| (t statistics) | (2.97) | (3.15) | (3.09) | (2.82) |

**Panel C: Friday - Monday**

|  | **CAPM** | **FF3** | **Carhart4** | **FF5** |
| --- | --- | --- | --- | --- |
| Age | -.01366 | -.0127231 | -.01174876 | -.0117207 |
| (t Statistics) | (-3.84) | (-3.64) | (-3.69) | (-3.22) |
| O Score | -.0102427 | -.0098194 | -.007896 | -.0099104 |
| (t statistics) | (-3.45) | (-3.67) | (-3.92) | (-3.69) |

Table S1

Excluding Macroeconomics News Announcements Dates

Table S1 examines Quality minus Junk strategy returns of the portfolios prepared on the basis of Age anomaly & O score anomaly to invest on Friday and Monday. Panel A represents QMJ weekly returns on Friday, panel B represents QMJ weekly returns on Monday and panel C represents the QMJ strategy returns for Friday minus Monday. All panel consists of the alpha values based on Fama & French 5 factor model, Carhart 4 factor model, Fama & French 3 factor Model and Capital Assets Pricing Model. Portfolios are generated on the basis of Value weighted technique and alpha values are also adjusted for heteroskedasticity and autocorrelation.

**Table S2: Exclusion of firm specific news announcement dates**

**Panel A: Friday Quality minus Junk**

|  | **CAPM** | **FF3** | **Carhart4** | **FF5** |
| --- | --- | --- | --- | --- |
| Age | -.0098376 | -.0101738 | -.0099728 | -.0092861 |
| (t Statistics) | (-3.88) | (-3.71) | (-3.49) | (-3.77) |
| O Score | -.0068282 | -.0075382 | -.0062862 | -.0057926 |
| (t statistics) | (-4.71) | (-4.85) | (-4.55 ) | (-4.05) |

**Panel B: Monday Quality-Junk**

|  | **CAPM** | **FF3** | **Carhart4** | **FF5** |
| --- | --- | --- | --- | --- |
| Age | -.0082716 | -.0072971 | -.0081291 | -.008829 |
| (t Statistics) | (-4.71) | (-4.52) | (-4.43) | (-4.68) |
| O Score | -.004839 | -.0039829 | -.003121 | -.003825 |
| (t statistics) | (-5.91) | (-6.42) | (-6.19) | (-5.99) |

**Panel C: Friday - Monday**

|  | **CAPM** | **FF3** | **Carhart4** | **FF5** |
| --- | --- | --- | --- | --- |
| Age | -.001566 | -.002045 | -.001844 | -.000457 |
| (t Statistics) | (-2.75) | (-2.59) | (-2.51) | (-2.86) |
| O Score | -.00199 | -.00355 | -.003165 | -.001968 |
| (t statistics) | (-3.98) | (-4.09) | (-4.32) | (-3.87) |

Table S2

Excluding Firm Specific News Announcements Dates.

Table S2 examines Quality minus Junk strategy returns of the portfolios prepared on the basis of Age anomaly & O score anomaly to invest on Friday and Monday. Panel A represents QMJ weekly returns on Friday, panel B represents QMJ weekly returns on Monday and panel C represents the QMJ strategy returns for Friday minus Monday. All panel consists of the alpha values based on Fama & French 5 factor model, Carhart 4 factor model, Fama & French 3 factor Model and Capital Assets Pricing Model. Portfolios are generated on the basis of Value weighted technique and alpha values are also adjusted for heteroskedasticity and autocorrelation.
